# Coroner’s Inquests

**Published:** 1859-01

**Authors:** 


*Coroner's Inquests.*—By Mr. Redgrave's return, 21,802 inquests were held during 1856 in England and Wales, at a cost of £67,000, and nearly 10,000 of the deaths in question were not shown to have been violent. 265 persons were committed for trial in consequence of these investigations, 109 were found guilty of manslaughter and murder, and sixteen were hanged.

